# PHLPP2 stabilization by p27 mediates its inhibition of bladder cancer invasion by promoting autophagic degradation of MMP2 protein

**DOI:** 10.1038/s41388-018-0374-1

**Published:** 2018-06-21

**Authors:** Minggang Peng, Jingjing Wang, Dongyun Zhang, Honglei Jin, Jingxia Li, Xue-Ru Wu, Chuanshu Huang

**Affiliations:** 1 Nelson Institute of Environmental Medicine, New York University School of Medicine, Tuxedo, NY 10987 USA; 2 Department of Obstetrics and Gynecology, Union Hospital, Tongji Medical College, Huazhong University of Science and Technology, 1277 JieFang Avenue, Wuhan, 430022 China; 3 School of Laboratory Medicine and Life Science, Wenzhou Medical University, Wenzhou, Zhejiang 325035 China; 4 Departments of Urology and Pathology, New York University School of Medicine, New York, NY 10016 USA

## Abstract

Pleckstrin homology domain leucine-rich repeat protein phosphatase 2 (PHLPP2) is a tumor suppressor that catalyzes the de-phosphorylation of the AGC kinases, while p27 acts as a tumor suppressor that regulates cell cycle, apoptosis, and cell motility. Our previous studies have identified that PHLPP2 participates in inhibition of transformation of human bronchial epithelial cells following lung carcinogen B[a]P/B[a]PDE exposure. However, nothing was known about the association of p27 with regulation of PHLPP2 expression and the role of PHLPP2 in bladder cancer (BC) invasion. In our current studies, we demonstrated that PHLPP2 inhibited BC invasion through promoting MMP2 degradation via p62-mediated autophagy; and p27 expression was able to stabilize PHLPP2 protein by inhibiting protein degradation of Hsp90, which could directly bind to PHLPP2 and protect it from degradation. More in-depth studies discovered that stabilization of Hsp90 by p27 was mediated by calpain1 proteolysis system, whereas p27 inhibited calpain1 gene transcription by attenuating Jak1/Stat1 cascade in human invasive BC cells. Collectively, we for the first time revealed PHLPP2 downregulation in BCs and its participating in promotion of BC invasion, as well as novel role of p27 and mechanisms underlying its regulation of PHLPP2 protein degradation through Hsp90-dependent manner. Our findings improve our understanding of p27 and PHLPP2 roles and their crosstalk in regulation of BC invasion, which further contributes to improve the current strategy for invasive bladder cancer therapy.

## Introduction

Bladder cancer (BC) is one of the most lethal diseases in developed countries with approximately 75,000 cases and 16,000 deaths in the United States in 2015 [1], and muscle invasive bladder cancer (MIBC) characterized by rapid progression, metastasis, and poor prognosis, therefore is the leading cause of bladder cancer-related deaths [2–4]. Thus, a better understanding of the molecular mechanisms involved in the invasion ability of MIBC could contribute to the discovery of therapeutic targets, which is urgently needed in order to help the increasing number of bladder cancer patients.

Pleckstrin homology domain leucine-rich repeat protein phosphatases, including PHLPP1 and PHLPP2, have been identified as phosphatases with PH domains [5]. Generally, the PHLPP family members are considered as tumor suppressors in several types of cancer due to their ability to block growth factor-induced signaling pathway in cancer cells [6, 7]. PHLPP2 has been identified by our lab to inhibit lung carcinogenesis following B[a]P/B[a]PDE exposure [8]. Moreover, we demonstrate that PHLPP2 is involved in NFκB2-mediated inhibition of BC growth [9]. However, little is known whether PHLPP2 is involved in modulation of the invasion ability of bladder cancer.

p27^Kip1^ is a negative cell cycle regulatory gene that plays a central role in the transition from late G1 to S phase [10]. Although mutation of the p27 gene is rare in human cancers, decreased p27 protein levels are found in multiple kinds of cancer, including BCs, and are associated with poor prognosis of the BC patient [11–15]. Furthermore, decreased p27 and cyclin E have been associated with the progression of BC from a superficial to invasive phenotype, indicating the involvement of p27 in modulation of BC invasion [16].

Calpains, which are represented by two main ubiquitously expressed isoforms, calpain1 and calpian2, are proteolytic enzymes that belong to a family of the Ca^2+^-dependent proteases. Calpains are able to specifically degrade members of protein complexes that are required to regulate and elicit cell responses [17, 18]. Moreover, calpain1 was identified to interact directly with Hsp90 in human T cells [19].

Autophagy is found to be a lysosome-based degradation mechanism of cytosolic cargos during an adaptation of cells to starvation or other stimulation [20, 21]. p62/SQSTM1 is a member of the growing list of autophagic receptors characterized by the presence of a LC3-interacting region (LIR), which allows interaction with the autophagic machinery [22, 23]. Limited reports indicate that p62 liberates Beclin1, thus inducing autophagy [24]. Though autophagy has been widely studied, little is known about its role in BC invasion, and more evidence is needed to fully illustrate the interplay among p62, autophagy and BC invasion.

In the current study, we characterize a pathway that increases the invasion ability in BC, which involves the induction of PHLPP2 through a p27/JAK1/STAT1/CALPAIN1/Hsp90 cascade. Based on our findings, PHLPP2 may function as a tumor suppressor by activating p62 transcription and expression, which further induces autophagy and promotes MMP2 degradation.

## Results

### Downregulated p27 and PHLPP2 contributed to bladder cancer cell invasion

**Fig. 1:**
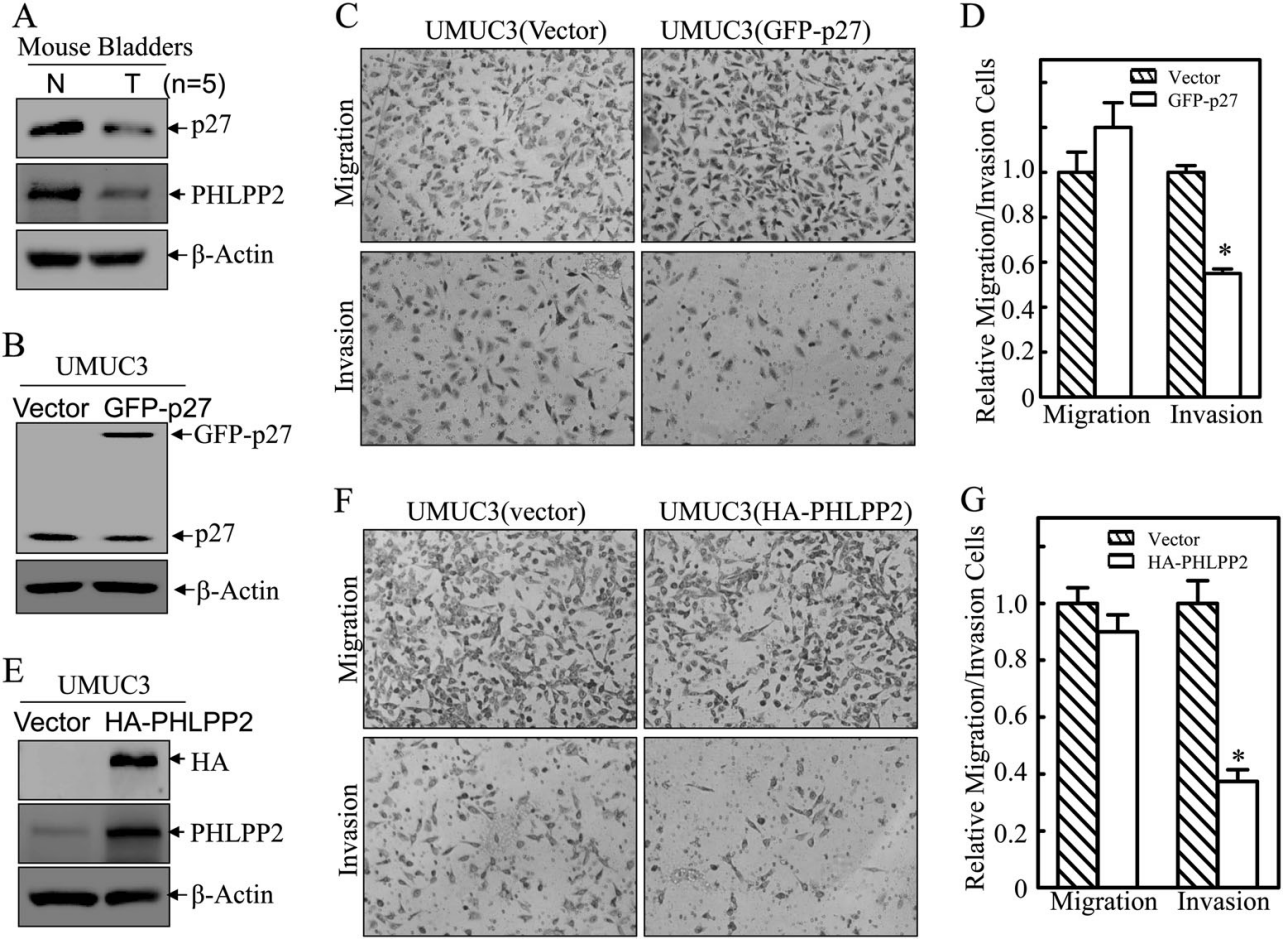
p27 and PHLPP2 inhibited human bladder cancer cell invasion. **a** Western blot (WB) was used to detect the protein level of p27 and PHLPP2 in mixed mouse bladder cancer tissue (*n* = 5). **b** Stable transfectants of human bladder cancer cells UMUC3(Vector) vs UMUC3(GFP-p27) were identified by western blot, β-Actin was used as protein loading control. **c** Invasion abilities of UMUC3(Vector) and UMUC3(GFP-p27) cells were determined using BD Biocoat^TM^ matrigel^TM^ invasion chamber. The migration ability was determined using the empty insert membrane without the matrigel, while the invasion ability was evaluated using the same system except that the matrigel was applied. **d** The invasion ability was normalized to the insert control according to the manufacturer’s instruction. The symbol (*) indicates a significant inhibition (*P* < 0.05). **e** Stable transfectants of UMUC3(Vector) vs UMUC3(HA-PHLPP2) were identified by western blot, β-Actin was used as protein loading control. **f** Invasion abilities of UMUC3(Vector) and UMUC3(HA-PHLPP2) cells were determined using the same method as described above. **g** The invasion rate between UMUC3(Vector) vs UMUC3(HA-PHLPP2) cells was calculated as described above. The symbol (*) indicates a significant inhibition (*P* < 0.05)

As tumor suppressors, both p27 and PHLPP2 have been found to be downregulated in human bladder cancer tissues [25]. To explore the role of both proteins in bladder tumor biology, we examined their expression levels in the mouse high invasive BC that were induced by exposure of mice to drinking water containing *N*-butyl-*N*-(4-hydroxybutyl) nitrosamine (BBN) as described in our previous studies [26]. As shown in Fig. 1a, expression levels of p27 and PHLPP2 were remarkably decreased in BC tissues as compared to normal bladder tissues. To explore the direct effects of p27 and PHLPP2 on BC invasion, we stably transfected GFP-27 and HA-PHLPP2 construct into human invasive BC UMUC3 cells, respectively. As shown in Fig. 1b–d, p27 overexpression resulted in a significant inhibition of UMUC3 invasive ability, while the migration ability was only slightly enhanced. Similarly, overexpression of PHLPP2 in UMUC3 cells (Fig. 1e) caused a dramatic reduction of cell invasion with minor effects on cell migration (Fig. 1f, g). Our results reveal that p27 and PHLPP2 have similar ability to inhibit cell invasion with little or no effect on cell migration in human invasive BC cells.

### p27 was required for PHLPP2 protein stabilization in human BC cells

**Fig. 2:**
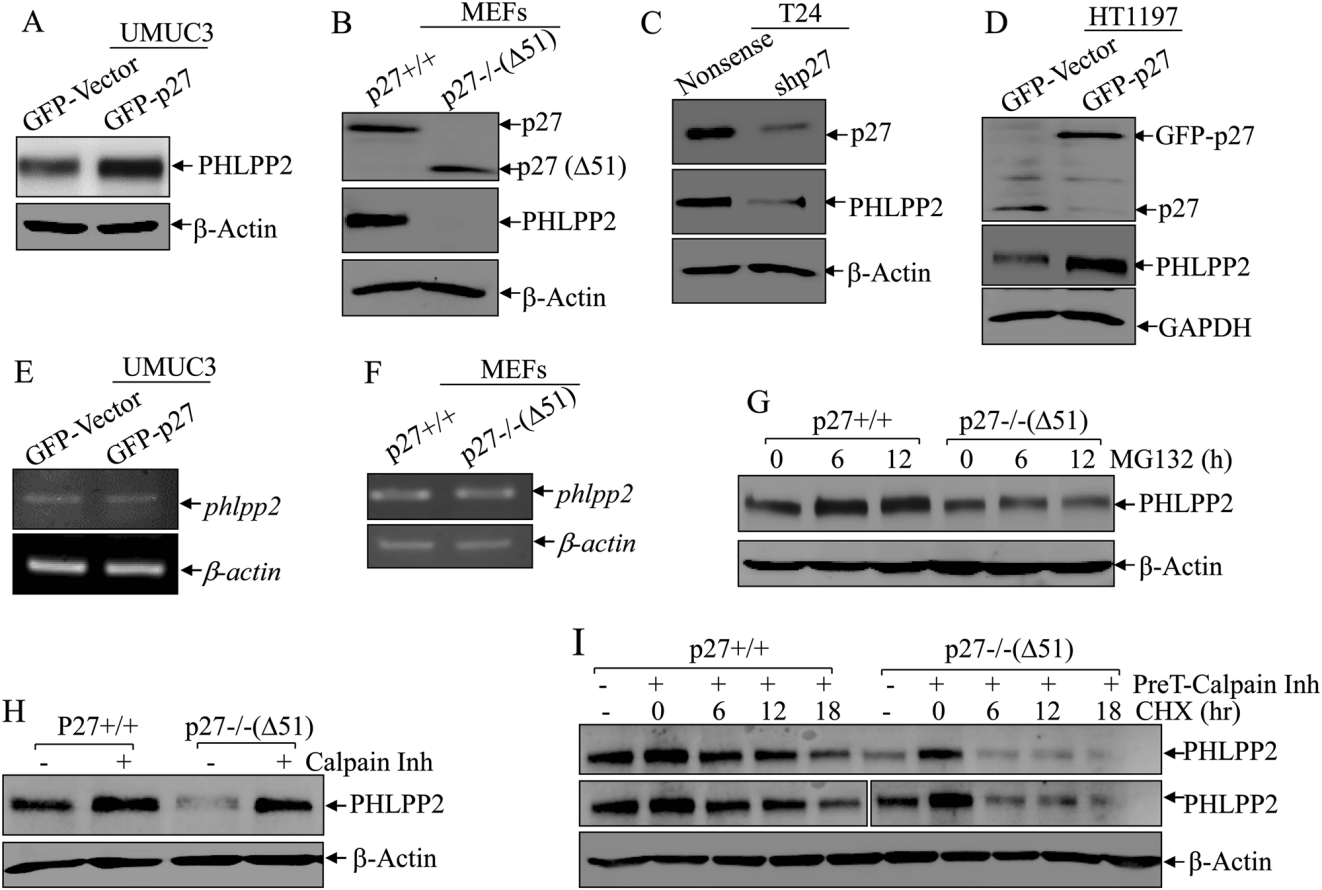
PHLPP2 was regulated by p27 at protein degradation level in Calpain-associated mechanism. **a**–**d** Western blot (WB) was used to detect the protein levels of PHLPP2 in UMUC3(Vector) vs UMUC3(GFP-p27) cells (**a**), p27+/+ vs p27−/−(Δ51) (**b**), T24(Nonsense) vs T24(shp27) (**c**), HT1197(Vector) vs HT1197(GFP-p27) (**d**). β-Actin or GAPDH was used as protein loading control. **e**, **f** RT-PCR was applied to compare the mRNA levels of phlpp2 in UMUC3(Vector) and UMUC3(GFP-p27) cells (**e**), and p27+/+ vs. p27−/−(Δ51) (**f**). β-actin was used as internal control. **g** p27+/+ and p27−/−(Δ51) cells were treated with MG132 for the indicated times. The cell extracts were then subjected to western blot to analyze PHLPP2 protein accumulation among the indicated cells. β-Actin was used as protein loading control. **h** p27+/+ and p27−/−(Δ51) cells were treated with Calpain inhibitor for 12 h. The cell extracts were then subjected to western blot analyses of PHLPP1 protein accumulation among the indicated cells. β-Actin was used as protein loading control. **i** p27+/+ and p27−/−(Δ51) cells were pre-treated with Calpain inhibitor for 12 h and the treated cells were subjected to protein degradation assay in presence of CHX and absence of Calpain inhibitor for the indicated time points. The cell extracts were then subjected to Western Blot to analyze PHLPP2 protein degradation rates between the indicated cells. β-Actin was used as protein loading control

Based on the similar inhibitory effects on BC cell invasion exhibited by both p27 and PHLPP2, we sought to determine whether there might be a crosstalk between the two tumor suppressors. To test this, we first checked PHLPP2 protein expressions in UMUC3(GFP-p27) and its scramble vector control transfectant. As shown in Fig. 2a, p27 overexpression markedly increased PHLPP2 protein abundance. We tested this finding in p27−/−(Δ51) mouse embryo fibroblasts (MEFs), which lack 51 amino acids of the N-terminal portion of p27 in comparison to p27+/+ MEFs. Consistently, PHLPP2 levels were dramatically reduced in p27 knockout cells (Fig. 2b). Moreover, knockdown of p27 in human BC T24 cells, T24(shp27), also resulted in a similar reduction of PHLPP2 expression (Fig. 2c), while overexpression of p27 in human BC HT1197 cells, HT1197(GFP-p27), increased PHLPP2 expression (Fig. 2d). Above results consistently demonstrate that p27 was required for PHLPP2 protein expression in human BC cells.

To examine the mechanism of p27 upregulation of PHLPP2, we compared the mRNA expressions of PHLPP2 in UMUC3(GFP-Vector) vs. UMUC3(GFP-p27) cells and in p27+/+ vs. p27−/−(Δ51) MEFs. No distinguishable difference at mRNA level was observed for either cell types of cells (Fig. 2e, f), indicating that PHLPP2 might be regulated at levels of either protein degradation or translation. To test these possibilities, MG132 and calpain inhibitor, two protein degradation inhibitors, were employed. Treatment of p27−/−(Δ51) cells with MG132, which blocks proteasome-mediated protein degradation, did not show restorative effect on PHLPP2 protein although it accumulated PHLPP2 protein in p27+/+ cells in (Fig. 2g), indicating that proteasome-dependent pathway is not involved in p27-mediated PHLPP2 protein expression. Next, treatment of p27−/−(Δ51) cells with calpain inhibitor (Calpain Inh), a competitive inhibitor of the Ca2+-dependent neutral cysteine protease, exhibited an remarkable positive effect on PHLPP2 protein accumulation (Fig. 2h), revealing that protease calpain might participate p27 regulation of PHLPP2 abundance in human BC cells. Therefore, we further assessed the PHLPP2 protein degradation rates in presence of CHX for inhibition of new protein synthesis between p27+/+ and p27−/−(Δ51) cells, both of which were pretreated with or without calpain inhibitor (PreT-Calpain Inh). As expected, PHLPP2 degraded much faster in CHX-treated p27−/−(Δ51) cells (half-life: 2.0 h) than those in CHX-treated p27+/+cells (half-life: 5.2 h) (Fig. 2i), demonstrating that p27 positively regulates PHLPP2 protein stability in calpain proteolysis-dependent axis.

### Hsp90 interacted with PHLPP2 to protect it from protein degradation

ERK has been reported to directly enhance calpain-mediated protein proteolysis [27]. To evaluate whether ERK is involved in the p27 regulation of PHLPP2 degradation, we evaluated the levels of phosphorylated ERK1/2 at T202/Y204. The p-ERK levels were inhibited by ectopic expression of p27, UMUC3(GFP-p27) and HT1197(GFP-p27), as compared with its scramble vector transfectants, though total protein levels were not changed (Fig. S1A,B). Consistently, p-ERK levels were enhanced in p27-deficient cells, p27−/−(Δ51) MEFs (Fig. S1C) and T24(shp27) cells (Fig. S1D). These results suggest that p27 expression exhibits an inhibitory effect on ERK activation. Moreover, we also introduced the plasmid HA-rERK2 K52R, which expresses a dominant negative mutant of ERK, into p27−/−(Δ51) cells, and the results exhibited that inhibition of ERK activity by its mutant resulted in upregulation of PHLPP2 protein (Fig. S1E) with no observed effect on PHLPP2 protein degradation (Fig. S1F). Collectively, above results rule out a primary role of ERK in mediating the effect of p27 on regulation of PHLPP2 protein stability.

**Fig. 3:**
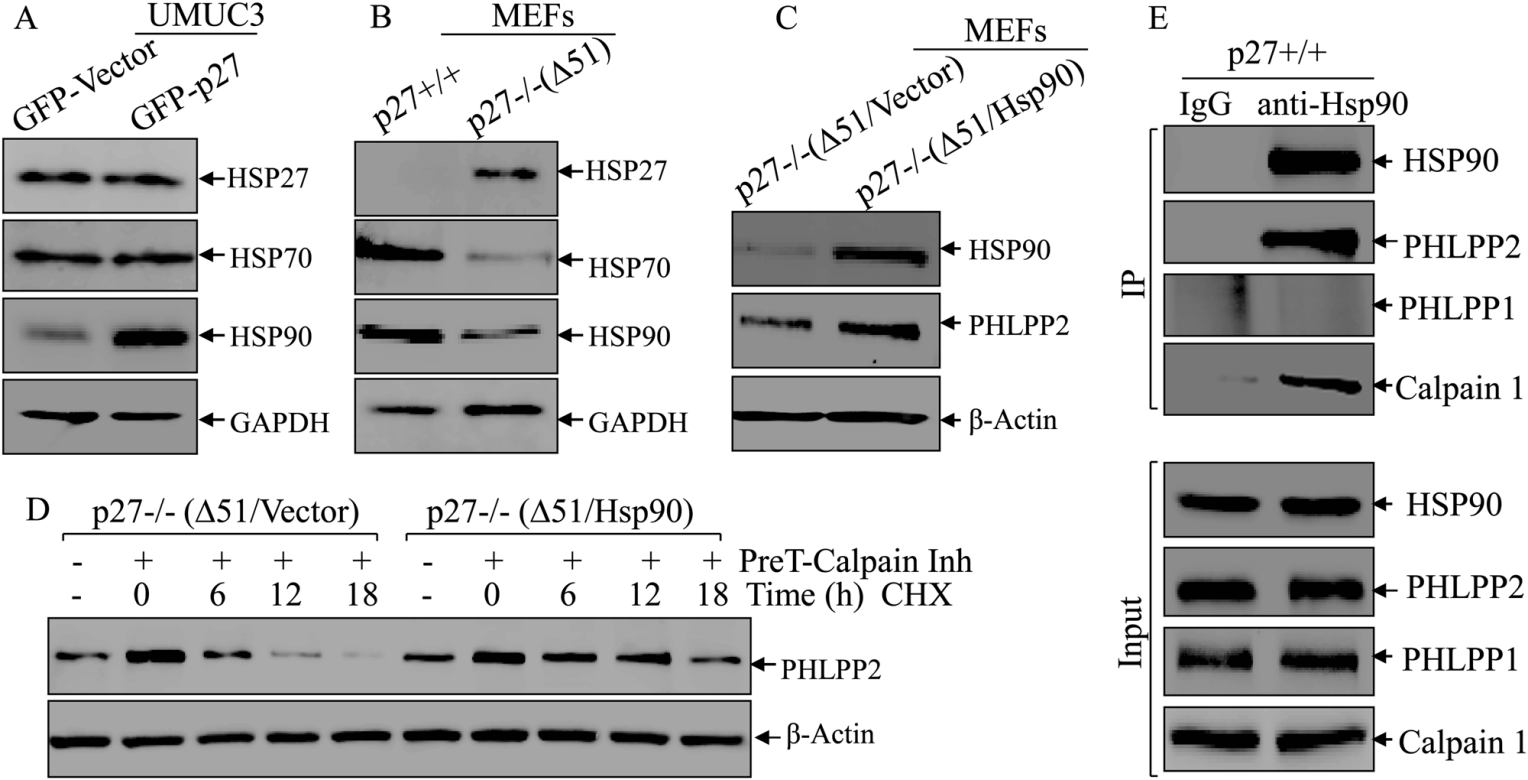
Hsp90 interacted with PHLPP2 and prevented it from degradation. **a**, **b** Western blot was used to detect the protein level of Hsp27, Hsp70, and Hsp90 in UMUC3(Vector) vs UMUC3(GFP-p27) cells (**a**), p27+/+ vs p27−/−(Δ51) cells (**b**). GAPDH was used as protein loading control. **c** Western blot was used to identify the protein level of Hsp90 in p27−/−(Δ51) cells stably transfected with ectopic Hsp90. PHLPP2 protein was evaluated in the indicated cells, β-Actin was used as protein loading control. **d** p27−/−(Δ51) and p27−/−(Δ51/Hsp90) cells were pre-treated with Calpain inhibitor for 12 h and the cells were then subjected to protein degradation assay in the presence of CHX and absence of Calpain inhibitor for the indicated time periods. The cell extracts were subjected to western blot to analyze PHLPP2 protein degradation rates among the indicated cells. β-Actin was used as protein loading control. **e** IP assay, described in “Materials and methods”, was applied to see the direct binding of Hsp90 and its substrates. We used anti-Hsp90 antibody to pulldown the proteins that could bind to Hsp90, and WB was used to detect the protein abundance of PHLPP2, PHLPP1 and Calpain1

Our previous studies indicate that heat shock proteins acts as a chaperon protein for stabilization of their bound proteins [28, 29]. To test this possibility, we examined a potential involvement of the heat shock proteins (Hsp27, Hsp70, and Hsp90) in p27 regulating PHLPP2 stability. Hsp27 and Hsp70 levels were not affected by p27 overexpression in UMUC3 (GFP-p27) cells as compared to UMUC3(GFP-Vector) cells, whereas Hsp90 was markedly increased in UMUC3(GFP-27) cells (Fig. 3a). Consistently, p27 deletion resulted in a greatly increased Hsp27 expression, while Hsp70 and Hsp90 were decreased in p27−/−(Δ51) as compared with that in p27+/+ cells (Fig. 3b). Thus, we anticipated that Hsp90 might be involved in p27 stabilization of PHLPP2. To address this possibility, we overexpressed Hsp90 in p27−/−(Δ51) cells, and the results showed that Hsp90 overexpression increased PHLPP2 protein expression (Fig. 3c). Furthermore, a PHLPP2 degradation assay in p27−/−(Δ51) (Vector) and p27−/− (Δ51) (Hsp90) cells demonstrated that ectopic expression Hsp90 stabilized PHLPP2 protein by preventing it from degradation (half-life: 2.3 h in p27−/−(Δ51/Vector) cells vs. 4.8 h in p27−/−(Δ51/Hsp90) cells (Fig. 3d). In addition, the results obtained from an immunoprecipitation assay showed a direct binding of Hsp90 with PHLPP2 and calpain1, but not PHLPP1 (Fig. 3e). Taken together, our results demonstrate that Hsp90 specifically interacts with PHLPP2 to prevent its degradation.

### Calpain1, but not calpain2, was responsible for Hsp90 degradation in p27-deleted cells

**Fig. 4:**
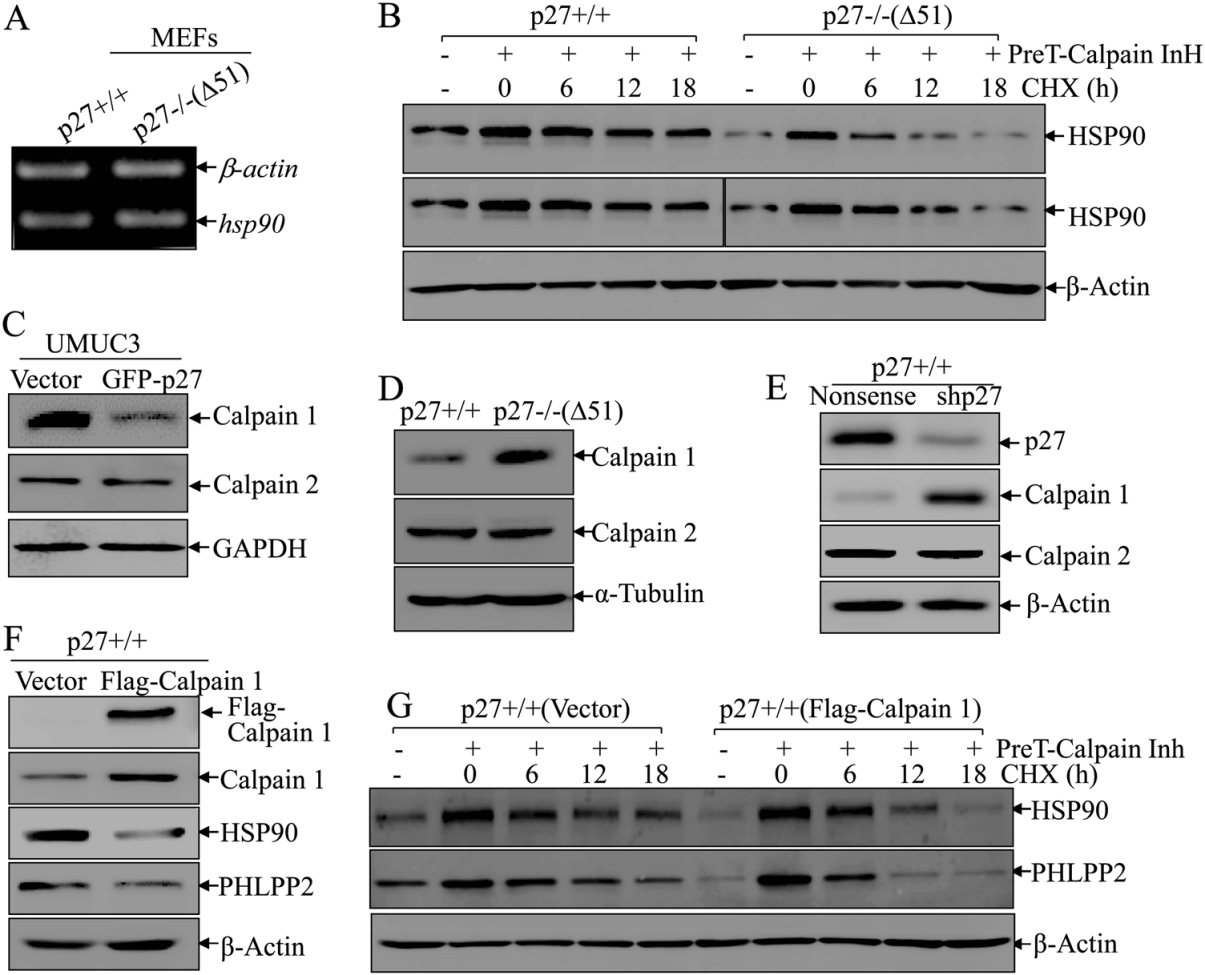
Calpain1 was responsible for p27 deletion-mediated Hsp90 degradation. **a** RT-PCR was applied to compare the mRNA levels of hsp90 between p27+/+ and p27−/−(Δ51) cells, β-actin was used as internal control. **b** p27+/+ and p27−/−(Δ51) cells were pre-treated with Calpain inhibitor for 12 h and the cells were then subjected to protein degradation assay in the presence of CHX and absence of Calpain inhibitor for the indicated time points. The cell extracts were then subjected to western blot to analyze Hsp90 protein degradation rates among the indicated cells. β-Actin was used as protein loading control. **c**–**e** Western blot was used to detect the protein levels of Calpain1 and Calpain2 in UMUC3(Vector) *vs* UMUC3(GFP-p27) cells (**c**), p27+/+ vs p27−/−(Δ51) cells (**d**) and p27+/+ (Nonsense) vs its p27+/+ (shp27) cells (**e**). α-Tubulin or β-Actin was used as protein loading control. **f** Western blot was used to identify the protein level of Calpain1 in p27+/+ cells stably transfected with ectopic Flag-Calpain1. Hsp90 and PHLPP2 protein were evaluated in the indicated cells, and β-Actin was used as protein loading control. **g** p27+/+ (Vector) and p27+/+ (Flag-Calpain1) cells were pre-treated with Calpain inhibitor for 12 h and the cells were then subjected to protein degradation assay in the presence of CHX and absence of Calpain inhibitor for the indicated time points. Western blot was used to analyze Hsp90 and PHLPP2 protein degradation rates among the indicated cells. β-Actin was used as protein loading control

To characterize the detailed mechanism by which p27 promotes Hsp90 protein expression, we further examined hsp90 mRNA levels between p27+/+ and p27−/−(Δ51) cells and there was no observable differences in Hsp90 mRNA levels (Fig. 4a), suggesting that Hsp90 is regulated by p27 at protein level. Therefore, we performed a degradation assay. Hsp90 degraded much faster in p27 deletion cells (half-life: 4.8 h) in comparison in p27 wild-type cells (half-life: 10.8 h) (Fig. 4b). Given the results from our immunoprecipitation assay (Fig. 3e) and those of another group suggesting that Hsp90 can directly interact with calpain1 in human T lymphocytes [19], we anticipate that calpain1 may mediate Hsp90 protein degradation. This notion was greatly supported by the results shown in Fig. 4c that calpain 1 was dramatically decreased while calpain 2 was not notably changed in UMUC3(GFP-p27) cells compared to their vector control transfectant. Consistently, p27 deficient in p27−/−(Δ51) led to an markedly increase in calpain 1 protein expression with no observable effect on calpain2 as compared to p27+/+ cells (Fig. 4d). Moreover, calpain 1 was also increased upon p27 knockdown in p27+/+ (shp27) versus p27+/+ (Nonsense) cells, while calpain 2 showed no obvious change (Fig. 4e). These results reveal that p27 specifically regulates Calpain 1, but not Calpain 2 in the intact cells, which may be involved in p27 modulation of expression of Hsp90 and PHLPP2 in human BC cells. To evaluate whether calpain1 is responsible for Hsp90 degradation, we introduced Flag-calpain1 into p27+/+ cells. Expression of Hsp90 and PHLPP2 was decreased upon calpain1 overexpression (Fig. 4f). In addition, we performed protein degradation assays in p27+/+ (Flag-calpain1) and p27+/+ (Vector) cells. As shown in Fig. 4g, degradation rates of Hsp90 and PHLPP2 was much faster in p27+/+ (Flag-calpain 1) (half-life: 4.3 and 2.4 h) in comparison to p27+/+ (Vector) cells (half-life: 10.0 and 5.4 h), revealing that calpain 1 is critical for promoting Hsp90 degradation and subsequent reducing PHLPP2 protein stability in human BC cells.

### Phosphorylation of Stat1 at Tyr701 by Jak1 mediated the transcriptional upregulation of calpain1

**Fig. 5:**
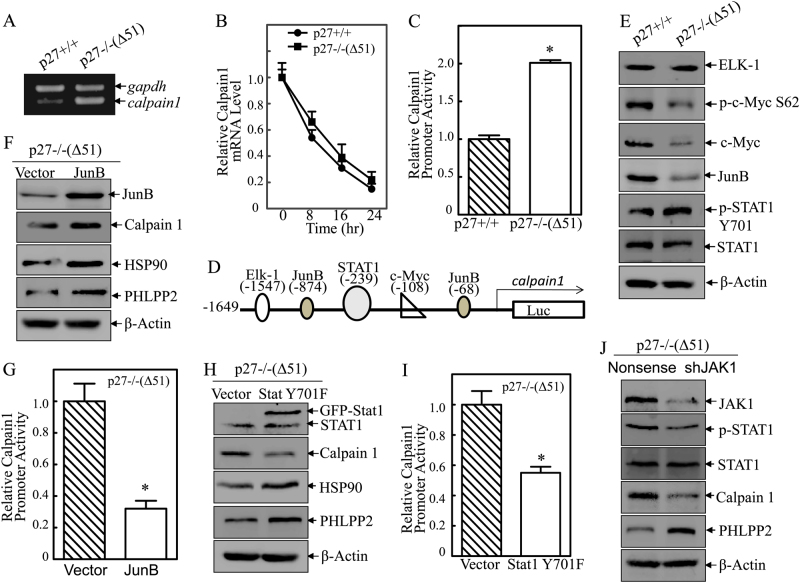
Jak1-mediated phosphorylation of Stat1 at Tyr701 was responsible for transcription activation of calpain1. **a** RT-PCR was applied to compare the mRNA levels of calpain1 between p27+/+ and p27−/−(Δ51) cells, and gapdh was used as internal control. **b** Calpain1 mRNA stabilities were evaluated by real-time PCR in presence of Act D in p27+/+ and p27−/−(Δ51) cells and the results were presented as relative calpain1 mRNA expression. **c** Mouse calpain1 promoter-driven luciferase reporter was co-transfected together with pRL-TK into p27+/+ and p27p27−/−(Δ51) cells, respectively. Twenty-hour hours post transfection, the transfectants were extracted to evaluate the luciferase activity. TK was used as internal control. The results were presented as relative calpain1 promoter activity, and each bar indicates a mean ± SD from three independent assays. The symbol (*) indicates a significant difference (*P* < 0.05). **d** The description of calpain1 promoter with the binding sites of potential transcription factors. **e** Western blot was used to detect the protein level of Elk-1, JunB, p-Stat1 Y701, Stat1, p-c-Myc S62, and c-Myc in p27+/+ vs p27−/−(Δ51) cells. β-Actin was used as protein loading control. **f** Western blot was used to determine the expression of JunB, Calpain1, Hsp90 and PHLPP2 in and p27−/−(Δ51/JunB) cells. β-Actin was used as protein loading control. **g** Mouse calpain1 promoter-driven luciferase reporter was co-transfected together with pRL-TK into p27−/−(Δ51) and p27−/−(Δ51/JunB) cells, respectively. Luciferase activity was evaluated as mentioned above. The results were presented as relative calpain1 promoter activity, and each bar indicates a mean ± SD from three independent assays. The symbol (*) indicates a significant difference (*P* < 0.05). **h** Western blot was used to determine the expression of Stat1 Y701F, Calpain1, Hsp90, and PHLPP2 in and p27−/−(Δ51/Stat1 Y701F) cells. β-Actin was used as protein loading control. **i** Mouse calpain1 promoter-driven luciferase reporter was co-transfected together with pRL-TK into p27−/−(Δ51) and p27−/−(Δ51/Stat1 Y701F) cells, respectively. Luciferase activity was evaluated as mentioned above. The results were presented as relative calpain1 promoter activity, and each bar indicates a mean ± SD from three independent assays. The symbol (*) indicates a significant difference (*P* < 0.05). **j** Western blot was used to identify the knockdown efficiency of Jak1 in p27−/−(Δ51) cells, and the protein levels of p-Stat1, Stat1, Calpain1 and PHLPP2 in p27−/−(Δ51) (shJak1) cells. β-Actin was used as protein loading control

To determine the mechanism of calpain1 upregulation by p27, we evaluated the mRNA expression of calpain1 in p27+/+ and p27−/−(Δ51) cells. Calpain1 mRNA was significantly increased upon p27 deletion (Fig. 5a), suggesting that calpain1 is regulated by p27 at the mRNA level, which could be either through mRNA stability or transcription. To test these possibilities, we determine the calpain 1 mRNA degradation rates in presence of Act D to block new mRNA transcription between p27+/+ and p27−/−(Δ51) cells. The results exhibited no significant difference in the calpain1 mRNA degradation between p27+/+ and p27−/−(Δ51) cells (Fig. 5b), excluding the p27 regulation of calpain 1 mRNA stability. To determine whether calpain 1 is regulated transcriptionally, a calpain 1 promoter-driven luciferase reporter was constructed and transfected into p27+/+ and p27−/−(Δ51) cells. As shown in Fig. 5c, calpain 1 promoter transcriptional activity was significantly higher in p27−/−(Δ51) cells compared to p27+/+ cells, demonstrating that p27 regulates calpain1 mRNA transcription.

To assess the mechanism of p27 transcriptional regulation of calpain 1, the potential transcription factors that are predicted to bind to the calpain1 promoter (Fig. 5d) were evaluated. Elk-1 showed no changes between p27+/+ and p27−/−(Δ51) cells, while the levels of c-Myc, p-c-Myc at Ser62 and Jun B were decreased and the levels of p-Stat1 at Tyr701 were increased in p27 deletion cells (Fig. 5e). Because Jun B typically functions as a transcription inhibitor [30] and Stat1 as an activator [31], we first examined both proteins as candidates for the regulation of calpain1 transcription. To assess the role of Jun B, Jun B expression construct was co-transfected with calpain1 promoter-driven luciferase reporter into p27−/−(Δ51) and p27+/+ cells. Ectopic expression of Jun B significantly inhibited calpain1 promoter activity (Fig. 5f, g). However, expression of calpain1, Hsp90 and PHLPP2 protein was also increased upon Jun B overexpression (Fig. 5f), which contradicts to our former results. Therefore, it is unlikely that the decrease in Jun B in p27−/−(Δ51) cells mediates calpain1 expression.

To evaluate the potential role of Stat1 in p27 inhibition of calpain 1 transcription, a dominant-negative mutated form of Stat1 (Stat1 Y701F) and calpain 1 promoter-driven luciferase reporter were stably co-transfected into p27−/−(Δ51) and p27+/+ cells. STAT1 inhibition dramatically suppressed calpain1 transcription (Fig. 5h, i), which was accompanied by promotion of downstream Hsp90 and PHLPP2 protein expression (Fig. 5h). Jak1 is a well-established upstream kinase responsible for mediation of Stat phosphorylation and activation [32]. Therefore, we knocked down Jak1 in p27−/−(Δ51) cells and assessed its effects on Stat 1 phosphorylation, calpain1and PHLPP2 expression. As shown in Fig. 5j, knockdown of Jak1 attenuated Stat1 phosphorylation in p27−/−(Δ51), while the total Stat1 protein was not significantly altered (Fig. 5j). Furthermore, the decreased Stat1 phosphorylation by knockdown of Jak1 led to a remarkably decrease in calpain1 protein and increase in PHLPP2 protein expression. Taken together, these results suggest that the Jak1/Stat1 pathway promotes calpain1 transcription, which in turn leads to Hsp90 and PHLPP2 destabilization.

### PHLPP2 mediated autophagy to promote MMP2 degradation and in turn inhibited BC cell invasion

**Fig. 6:**
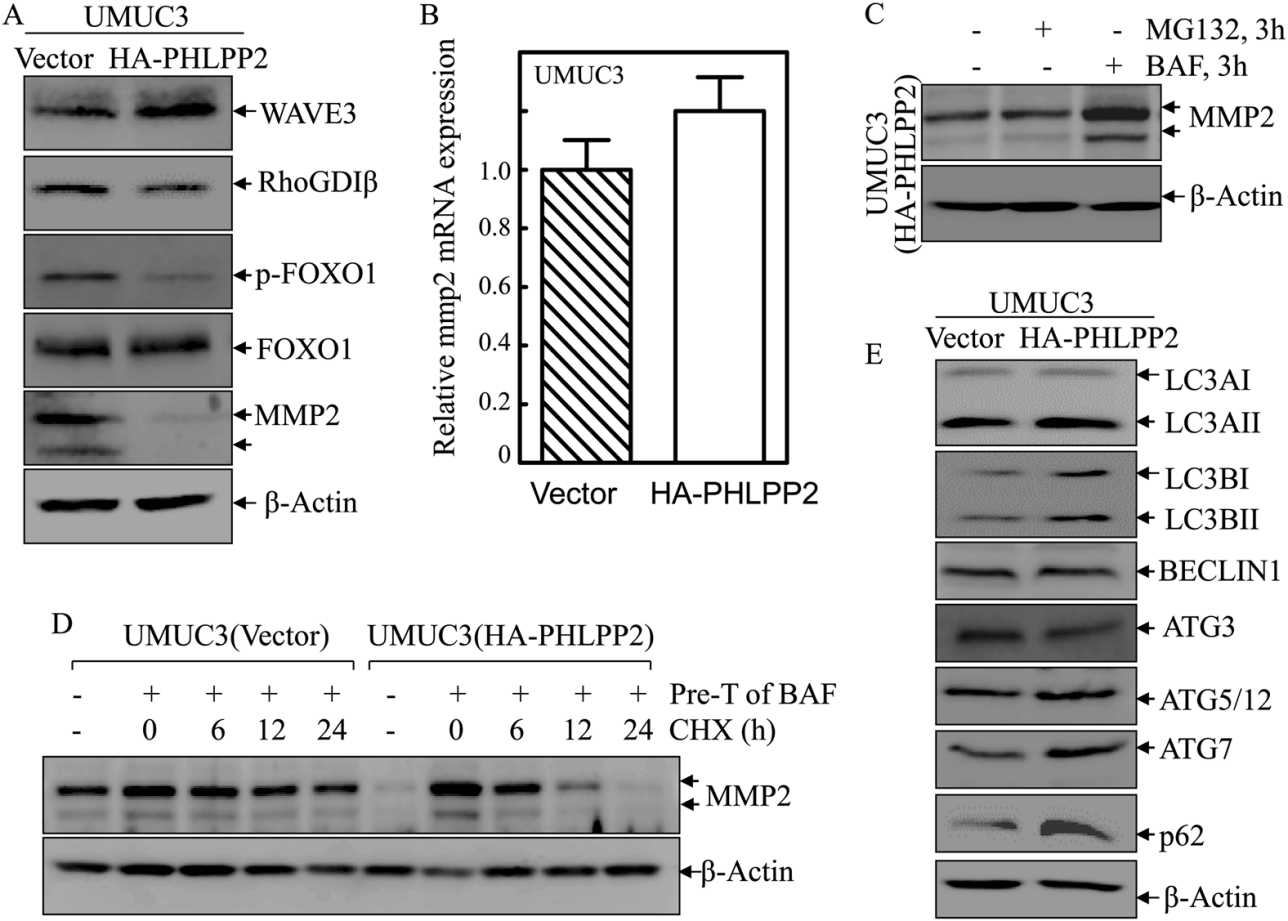
PHLPP2-mediated autophagy promoted MMP2 degradation. **a** Western blot was used to detect the protein levels of WAVE3, RhoGDIβ, p-FOXO1, FOXO1, and MMP2 in UMUC3(Vector) and UMUC3(HA-PHLPP2) cells. β-Actin was used as protein loading control. **b** Real-time PCR was applied to compare the mRNA levels of mmp2 in UMUC3(Vector) and UMUC3(HA-PHLPP2) cells. **c** Western blot was used to detect the protein levels of MMP2 in UMUC3(HA-PHLPP2) cells upon treatment of MG132 and Bafilomycin. β-Actin was used as protein loading control. **d** UMUC3(Vector) and UMUC3(HA-PHLPP2) cells were pre-treated with bafilomycin for 3 h and the cells were then subjected to protein degradation assay in presence of CHX for the indicated time points. Western blot was used to analyze MMP2 protein degradation rates among the indicated cells. β-Actin was used as protein loading control. **e** Western blot was used to detect the protein level of LC3A, LC3B, ATG3, ATG5/12, ATG7, Beclin1, and p62 in UMUC3(Vector) and UMUC3(HA-PHLPP2) cells; β-Actin was used as protein loading control

To elucidate the underlying mechanism of PHLPP2 inhibition of BC invasion, we evaluated several invasion-related proteins in BC cells that have been identified by the studies from our lab and others [26, 33–35]. Overexpression of PHLPP2 caused an increase in the level of Wave3 and decreases in the levels of RhoGDIβ, p-FOXO1, and most dramatically, MMP2 (Fig. 6a). Given the critical role of MMP2 in bladder cancer invasion, as verified in our previous study [36], these findings are consistent with the possibility that the inhibitory effect of PHLPP2 on BC invasion, therefore downregulation of MMP2 by PHLPP2 may play a major role in its inhibition of BC invasion. We next evaluated MMP2 mRNA expression in UMUC3(Vector) and UMUC3(HA-PHLPP2) cells, and the results did not show significantly different (Fig. 6b). To determine whether MMP2 is regulated at the protein degradation level by either proteasome or autophagy, we used MG132 and bafilomycin, to inhibit the protein degradation process. Interestingly, MG132 had no effect on MMP2 accumulation, while bafilomycin, an inhibitor of autophagy, caused a markedly MMP2 protein accumulation (Fig. 6c). The results indicate that autophagy is essential for MMP2 protein downregulation due to PHLPP2 overexpression. This notion was further supported by the results obtained from utilization of bafilomycin pretreatment to accumulate MMP2 protein and then evaluation of MMP2 protein degradation in presence of new protein synthesis inhibitor CHX. The results showed that MMP2 degraded much faster in UMUC3(HA-PHLPP2) cells than in UMUC3(Vector) cells (Fig. 6d). Consistently, autophagy markers, LC3BI and LC3BII expression was increased in UMUC3(HA-PHLPP2) accompanied with upregulated expression ATG7 and p62 and no observable alterations of ATG5/12 complex and Beclin1 (Fig. 6e), suggesting that ATG7 and p62 might be associated with PHLPP2-dependent upregulation of autophagy to promote MMP2 degradation in human BC cells.

### PHLPP2 promoted p62 transcription to mediate autophagy in human BC cells

**Fig. 7:**
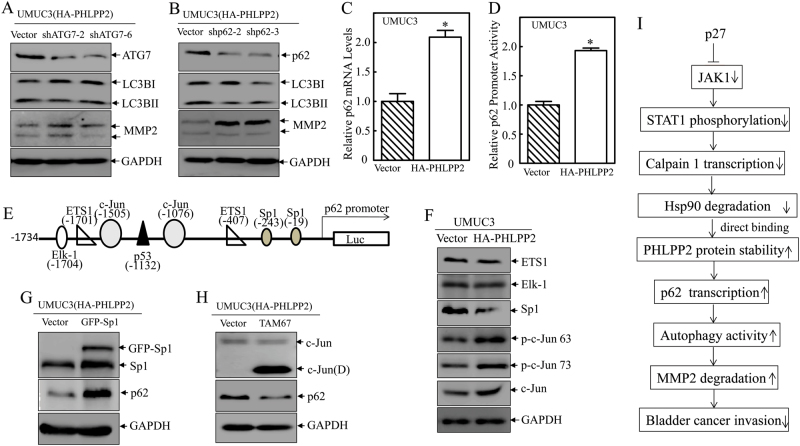
PHLPP2 promoted p62 transcription and in turn mediated autophagy in human BC cells. **a**, **b** Western blot was used to identify the knockdown efficiency of ATG7 (**a**) and p62 (**b**) in UMUC3(HA-PHLPP2) cells, as well as the protein levels of LC3BI, LC3BII, and MMP2. GAPDH was used as protein loading control. **c** Real-time PCR was applied to compare the mRNA levels of p62 in UMUC3(Vector) and UMUC3(HA-PHLPP2) cells. **d** Human p62 promoter-driven luciferase reporter was co-transfected together with pRL-TK into UMUC3(Vector) and UMUC3(HA-PHLPP2) cells. Twenty-four hours post transfection, the transfectants were extracted to evaluate the luciferase activity. TK was used as internal control. The results were presented as relative p62 promoter activity, and each bar indicates a mean ± SD from three independent assays. The symbol (*) indicates a significant difference (*P* < 0.05). **e** The diagram shows the transcription factors which could potentially bind to human p62 promoter region. **f** Western blot was used to detect the protein levels of ETS1, Elk-1, Sp1, p-c-Jun Ser63, p-c-Jun Ser73, and c-Jun in UMUC3(Vector) and UMUC3(HA-PHLPP2) cells. GAPDH was used as protein loading control. **g** Western blot was used to identify the overexpression efficiency of Sp1 in UMUC3(HA-PHLPP2) cells, and the protein level of p62 in UMUC3 (HA-PHLPP2) (shSp1) cells. GAPDH was used as protein loading control. **h** Western blot was used to identify the overexpression level of TAM67, and the protein level of p62 after TAM67 was stably transfected into UMUC3 (HA-PHLPP2) cells. GAPDH was used as protein loading control. **i** The mechanisms underlying p27 regulation of PHLPP2/MMP2 and BC invasion

To further explore the potential role of ATG7 and p62 in PHLPP2 upregulating autophagy and its dependent MMP2 degradation, the shRNA specifically targeting human ATG7 (shATG7) and human p62 (shp62) were stably transfected into UMUC3(HA-PHLPP2) cells, respectively. Knockdown of ATG7 only showed a slightly effect on LC3BII protein level or MMP2 expression level (Fig. 7a), whereas p62 knockdown led to a remarkably inhibition of autophagy as indicating the reduction of LC3BII accompanied with a remarkable increase in MMP2 protein abundance (Fig. 7b). Therefore, p62 promotes PHLPP2-mediated autophagy and MMP2 degradation in human BC cells.

To explore the mechanisms underlying PHLPP2 upregulation of p62, we examined p62 mRNA expression in UMUC3(Vector) and UMUC3(HA-PHLPP2) cells. The results demonstrated that PHLPP2 overexpression significantly increased p62 mRNA levels (Fig. 7c), which could be explained by either activation of transcription or inhibition of mRNA degradation. To distinguish these possibilities, the human p62 promoter-driven luciferase reporter was transfected into UMUC3(Vector) and UMUC3(HA-PHLPP2) cells and the effect of PHLPP2 on p62 promoter transcriptional activity was determined. As shown in Fig. 7d, p62 promoter transcriptional activity was greatly enhanced by overexpression of PHLPP2. Thus, the potential transcription factors that are predicted to bind human p62 promoter region was analyzed and shown in Fig. 7e. The results from evaluating the effect of PHLPP2 overexpression on these potential transcription factors indicated that ectopic expression of PHLPP2 did not show observable effect on ETS1 and Elk, whereas it exhibited an inhibition of Sp1, and promotion of p-c-Jun and total c-Jun expression in UMUC3 cells (Fig. 7f). To further determine whether Sp1 or c-Jun may be responsible for PHLPP2 mediating p62 transcription, we overexpressed GFP-Sp1 or a dominant negative mutant of C-Jun, TAM67, in UMUC3(HA-PHLPP2) cells, respectively. As shown in Fig. 7g, Sp1 overexpression showed a positively effect on p62 abundance, while c-Jun dominant-negative mutant exhibited a markedly inhibition of p62 expression (Fig. 7h). Collectively, our results demonstrate that c-Jun activation is critical for PHLPP2 promoting p62 transcriptional upregulation in human BC cells.

## Discussion

Invasive bladder cancer has been a persistent challenge for urologists and scientists for the past decades due to its poor prognosis, even when applying current therapeutic strategies [37]. To combat this deadly disease, comprehensive knowledge of the invasion mechanisms is urgently required. Loss of p27 has been associated with BC progression and poor prognosis [38]. However, the mechanisms of p27 regulation of BC invasion have not been elucidated. Here, we identified a novel pathway by which p27 inhibits BC cell invasion through another tumor suppressor, PHLPP2, in a MMP2-dependent manner (Fig. 7i). We, therefore for the first time, revealed a correlation between p27 and PHLPP2, which showed p27 stabilization of PHLPP2 protein through direct binding of Hsp90 and PHLPP2. Unlike p53, which is frequently mutated in human bladder cancer [39], p27 and PHLPP2 are functional, with rare mutations in human BCs. This observation suggests a promising opportunity by which p27 and PHLPP2 may serve as specific targets for the treatment of human invasive BCs.

The PHLPP family has been characterized as the phosphatases for three target molecules, Akt, PKC (protein kinase C), and S6K1 (ribosomal protein kinase 1), and thus they play important roles in various cellular processes based on these and a variety of targets that are further downstream [7, 40, 41]. Although new downstream targets are continually being identified; the PHLPP upstream regulator remains largely unknown. Here, we determined that p27 can stabilize PHLPP2 protein via inhibiting Calpain 1-mediated Hsp90 degradation, by which increases direct binding of Hsp90 with PHLPP2 protein for its stabilization. We further define that the upregulated PHLPP2 by p27 mediates C-Jun activation, in turn promotes C-Jun-regulated p62 transcription, and further leads to autophagy and autophagy-dependent MMP2 protein degradation, and consequently inhibits invasion of human BC cells. Therefore, we have revealed a pathway by which the classical tumor suppressor p27 and the newly identified p27 downstream PHLPP2 coordinately suppress BC invasion. Interestingly, our results suggest that the regulation of the two PHLPP isoforms by p27 occurs through different pathways and at different levels. Notably, we reveal for the first time that PHLPP2 is an Hsp90 binding protein, which can directly bind and stabilize PHLPP2, but not PHLPP1. Moreover, we revealed a role of Jak1/Stat1 axis in activating calpain1 transcription, indicating a novel relationship between the Jak/Stat pathway and the calpain proteolysis system.

The matrix metalloproteinases (MMPs) are a family of zinc-dependent proteolytic enzymes that are structurally and functionally related and are capable of degrading the extracellular matrix, thus promoting cancer cell invasion and metastasis [42]. In general, MMPs have been found to be highly expressed and/or with higher activity, in malignant cancers compared to normal or benign tissues [43]. Among them, MMP2 primarily hydrolyzes type IV collagen, the major structural component of the basement membrane [44]. Many studies have verified that MMP2 is critical in mediating BC invasion [36, 45], but the maintenance of MMP2 abundance has not been well studied. Based on our results here, overexpression of PHLPP2 significantly decreases MMP2 protein level, which is known to regulate BC cell invasion. Interestingly, in UMUC3(HA-PHLPP2) cells, MMP2 protein expression could be accumulated by treatment of cells with the autophagy inhibitor bafilomycin, but not by MG132, which targets the proteasome-dependent protein degradation pathway. In addition, overexpression of PHLPP2 greatly increased MMP2 degradation rates. Collectively, our study reveals a novel mechanism by which PHLPP2-initiated autophagy can promote MMP2 protein degradation in BC cells, thus inhibiting its invasion ability. To the best of our knowledge, this is the first report of a crosstalk between PHLPP2 and autophagy, as well as their regulation of MMP2 protein degradation.

Our further investigation revealed that PHLPP2 promotion of autophagy is dependent on p62. Though it is commonly accepted that p62 is degraded in the autophagy process as an autophagic adaptor [46], the effect of p62 on autophagy is still controversial and more detailed mechanisms are needed to elucidate its function [47]. p62 has been reported to interact with raptor, which is a core component of mTORC1, and to activate mTORC1 to inhibit autophagy [48]. Nevertheless, p62 and LC3 expression has been shown to be upregulated in colorectal cancer tissues, and knockdown of p62 significantly attenuates autophagy both in vitro and in vivo, indicating that p62 promotes autophagy [49]. In our current studies, knockdown of p62 in UMUC3(HA-PHLPP2) cells significantly decreased autophagy influx demonstrating that p62 is a target for PHLPP2 promotion of autophagy. We also determined that PHLPP2 can enhance expression of c-Jun, which binds to the p62 promoter region and activates its transcription. Therefore, we identify a new multi-component involved in PHLPP2-mediated autophagy that is dependent on p62.

In summary, our findings reveal a novel pathway by which p27 stabilizes PHLPP2 protein, which induces p62-dependent autophagy to promote MMP2 protein degradation and inhibit BC cell invasion. Hsp90 is demonstrated to act as a p27 downstream mediator to directly bind to PHLPP2 and in turn enhancing its protein stability in calpain1-dependent manner, while calpain 1 is regulated by the p27/Jak1/Stat1 cascade at the transcriptional level. These findings contribute to a better understanding of the crosstalk between p27 and PHLPP2 in suppressing BC invasion, which may facilitate the development of improved approach using PHLPP2 enzyme as a target for treatment of human invasive BCs in the future.

## Materials and methods

### Cell lines and transfection

The 3T3 protocol-immortalized wild-type p27 (p27+/+) and p27-deficient (p27−/−(Δ51)) mouse embryonic fibroblasts (MEFs) were described in previous study [50], and were cultured in DMEM with 10% FBS. Human invasive bladder cancer cell line UMUC3 and T24 [36, 51] was cultured in DMEM with 10% FBS and DMEM/Ham’s F-12 (1:1) with 5% FBS, respectively, while human transitional bladder cancer HT1197 [52] was cultured in MEM with 10% FBS. Cell transfections were performed with PolyJet^TM^ DNA in Vitro Transfection Reagent (SignaGen Laboratories, Rockville, MD), according to the manufacturer’s instructions. For stable transfection, MEFs were subjected to selection with hygromycin B (200 μg/mL) or puromycin (2.0 μg/mL) depending on the different antibiotic resistance of the plasmids transfected. Cells surviving from the antibiotic selection were pooled as mass stable transfectants. UMUC3 and T24 transfectants were selected by puromycin (0.4 μg/mL).

### Reagents, antibodies, and plasmids

Chemicals of Actinomycin D (Act D) (sc-200906), CHX (sc-3508), MG132 (sc-201270), and bafilomycin (sc-201550) were purchased from Santa Cruz (Dallas, TX). The calpain inhibitor was purchased from Cayman Chemical (Ann Arbor, MI). TRIzol reagent and SuperScript™ First-Strand Synthesis system were bought from Invitrogen (Grand Island, NY). The Dual Luciferase Assay kit was bought from Promega (Madison, WI). Antibodies specific against HA, LC3A, LC3B, ATG3, ATG5, ATG7, ATG12, Beclin1, calpain1, calpain2, Hsp27, Hsp70, Hsp90, Elk1, ETS1, p-Stat1 Y701, Stat1, Jak1, WAVE3, RhoGDIβ, p-ERK T202/Y204, ERK1/2, p-FOX01, FOXO1, p-c-Myc Ser 62, c-Myc, p-c-Jun Ser 63, p-c-Jun Ser 73, c-Jun, and c-Jun(D), were purchased from Cell Signaling (Beverly, MA). Antibodies specific for p27, Jun B, Sp1, MMP2, β-Actin, and α-Tubulin were purchased from Santa Cruz (Dallas, TX). Specific antibodies against PHLPP1and PHLPP2 were bought from Bethyl Laboratories (Montgomery, TX). Specific antibodies against p62 and GAPDH were bought from Genetex (Invine, CA). The shRNAs that specifically targets human p27, p62, Atg7, mouse p27, Sp1, Jak1 and their nonsense control vector were purchased from Open Biosystems (GE, Pittsburgh, PA). The plasmid constructs of GFP-p27, Hsp90, JunB, HA-PHLPP2, GFP-STAT1 Y701F and HA-rERK K52R were described in our previous studies [8, 26, 29, 50, 52]. The FLAG-calpain1 construct was obtained from Addgene [53]. The plasmid containing luciferase reporter under control of human p62 gene promoter was described in our previous studies [8], while the plasmid containing luciferase reporter under control of mouse calpain1 promoter was constructed into PGL3-Basic vector using the primers: forward 5′-CCG CTC GAG CCA GAC CAC AGT GGG ACA-3′, reverse 5′-CCC AAG CTT CAA AAG ACA AGG AGG ATG-3′.

### Western blot analysis

Whole-cell extracts were prepared with the cell lysis buffer (10 mM Tris-HCl, pH 7.4, 1% SDS, and 1 mM Na_3_VO_4_) as described in our previous studies [54]. Protein extracts were subjected to western blot with the indicated primary antibodies, and probed with the AP-conjugated secondary antibody together with the enhanced chemifluorescence system as described in a previous report [54]. The images were acquired by scanning with the PhosphorImager Typhoon FLA 7000 (GE, Pittsburgh, PA). The densitometry analyses of the specific protein degradation were performed using software Image J (NIH, Bethesda, MD) to obtain the protein half-life, which is calculated using the formula described in a published article [55].

### RT-PCR

Total RNA was extracted using the TRIzol reagent (Invitrogen, Grand Island, NY) as described by the manufacturer’s instructions and in our previous studies [36]. Five micrograms of total RNA was used for first-strand cDNA synthesis with oligdT primer by SuperScript^TM^IV First-Strand Synthesis System (Invitrogen, Grand Island, NY) described in our previous studies [36]. Specific primers (Invitrogen, Grand Island, NY) used for PCR amplification were displayed in supplementary materials (Table 1).

### Luciferase reporter assay

Calpain1 promoter luciferase reporter, human p62 luciferase reporter and pRL-TK, were stably transfected into p27+/+, p27−/−(Δ51), and UMUC3 (HA-PHLPP2) cells with specific antibiotic selection as described in above transfections. Luciferase activity was determined using the Dual Luciferase Assay system kit purchased from Promega (Madison, WI, USA) [56]. The results were normalized to internal TK activity and the results were expressed as mean ± SD error from three independent experiments.

### Co-immnunoprecipitation assay

p27+/+ cells were cultured in 10 cm dishes until the cell concentration reached 70–80%. Culture medium was replaced by DMEM containing 0.1% FBS for 12 h, cells were collected and lysed in 1× Cell Lysis Buffer (Cell Signaling Technology) containing protease inhibitors (Roche, Branchburg, NJ) followed by brief sonication. Cell extracts were incubated with Anti-Hsp90 or anti-mouse IgG (Cell Signaling Technology) with protein A/G agarose beads overnight at 4 °C. Following a brief centrifuge, the beads were washed three times with 1× Cell Lysis Buffer, and the bound proteins were eluted by 2× SDS sample buffer (Cell Signaling Technology, 56383) and subjected to western blot assay [57].

### Cell invasion assay

The invasion assay was performed according to the manufacturer’s instruction using the invasion kit purchased from BD falcon (Becton, Dickinson and Company, 354480). After incubation for 24 h, the cells both on the inside and outside of the chamber were fixed with 3.7% formalin for 2 min, washed twice with PBS, then transferred to 100% methanol for 20 min, washed twice again, then finally the cells were stained by Giemsa (1:20 diluted with PBS) at room temperature for 15 min in dark. After the staining, washed twice, then the non-invaded cells were scraped off with a cotton swab (PBS wetted). The photographs were taken under an Olympus microscopy DP71, and the number of the cells was calculated by the software “image J”.

### Statistical analysis

The Student’s *t*-test was used to determine the significance between treated and untreated group. The results are expressed as mean ± SD from at least three independent experiments. *P* < 0.05 was considered to be a significant difference between compared groups.

## Electronic supplementary material

Figure S1

Table 1
