# Supplementary figures and images for: PHLPP2 stabilization by p27 mediates its inhibition of bladder cancer invasion by promoting autophagic degradation of MMP2 protein
Source: Oncogene. 2018 Jun 21;37(43):5735–48. doi: 10.1038/s41388-018-0374-1 (PMC6202328; doi:10.1038/s41388-018-0374-1)

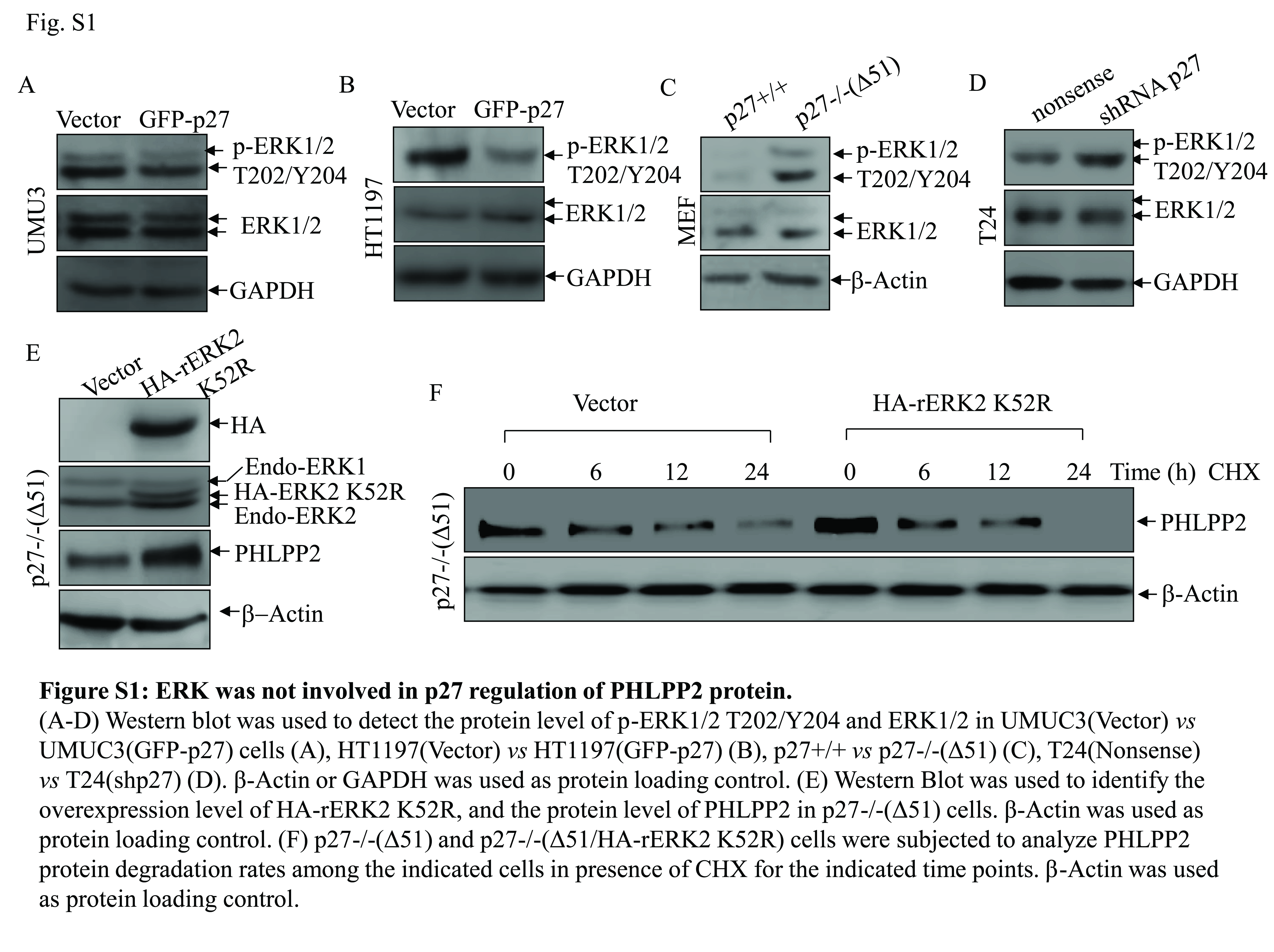

Supplement: Supplementary file 1 — Figure S1 [file 41388_2018_374_MOESM1_ESM.tif]
