# Supplementary material for: PHLPP2 stabilization by p27 mediates its inhibition of bladder cancer invasion by promoting autophagic degradation of MMP2 protein
Source: Oncogene. 2018 Jun 21;37(43):5735–48. doi: 10.1038/s41388-018-0374-1 (PMC6202328; doi:10.1038/s41388-018-0374-1)
Supplement: Supplementary file 2 — Table 1 [file 41388_2018_374_MOESM2_ESM.docx]

Table 1, Primers that are synthetized for the experiments (F: Forward; R: Reverse).

| \| *mouse phlpp2-F* \| 5’- AGG TTC CTG AGC ATC TCT TC -3’ \| \| --- \| --- \| \| *mouse phlpp2-R* \| 5’- GTT CAG GCC CTT CAG TTG AG -3’ \| \| *mouse hsp90-F* \| 5’- AGT AAA CTG GAC TCG GGG AA-3’ \| \| *mouse hsp90-R* \| 5’- CCT GCA AAG CCT CCA TGA AG -3’ \| \| *mouse calpain1-F* \| 5’- TGC CGC AGC ATG GTG AAC CTC -3’ \| \| *mouse calpain1-R* \| 5’- GAC TTG TCC AGG TCA AAC TTC C -3’ \| \| *human mmp2-F* \| 5’- CAA GTG GGA CAA GAA CCA GA -3’ \| \| *human mmp2-R* \| 5’- CCA AAG TTG ATC ATG ATG TC -3’ \| \| *human p62-F* \| 5’- GAG AGT GTG GCA GCT GCC CT -3’ \| \| *human p62-R* \| 5’- GGC AGC TTC CTT CAG CCC TG -3’ \| \| *mouse β-actin-F* \| 5’- GAC GAT GAT ATT GCC GCA CT -3’ \| \| *mouse β-actin-R* \| 5’- GAT ACC ACG CTT GCT CTG AG -3’ \| \| *human gapdh-F* \| 5’- GAT GAT CTT GAG GCT GTT GTC -3’ \| \| *human gapdh-R* \| 5’- CAG GGC TGC TTT TAA CTC TG -3’ \| |
| --- | --- | --- | --- | --- | --- | --- | --- | --- | --- | --- | --- | --- | --- | --- | --- | --- | --- | --- | --- | --- | --- | --- | --- | --- | --- | --- | --- | --- |
